# Development of a predictive model for severe adverse outcomes following surgery for neonatal necrotizing enterocolitis: a nomogram study based on postoperative intestinal failure beyond 42 days and death

**DOI:** 10.3389/fped.2025.1670493

**Published:** 2025-12-02

**Authors:** Ying Wu, Hongxia Ren

**Affiliations:** 1 Department of Neonatal Surgery, Shanxi Children’s Hospital, Taiyuan, China; 2 Department of Pediatrics, Shanxi Medical University, Taiyuan, China

**Keywords:** neonatal necrotizing enterocolitis, intestinal failure, death, nomogram, outcomes

## Abstract

**Objective:**

To identify the risk factors for intestinal failure occurring beyond 42 days postoperatively or death in neonates with necrotizing enterocolitis (NEC), and to develop a nomogram for predicting the likelihood of these outcomes.

**Methods:**

A retrospective cohort study was conducted on neonates who underwent surgical intervention for NEC at Shanxi Children's Hospital between January 1, 2018 and December 31, 2023. According to clinical outcomes, the patients were classified into two groups: those who either developed intestinal failure occurring beyond 42 days postoperatively or died, and a control group without intestinal failure. Univariate analysis and LASSO regression were employed to identify the optimal predictive variables. These variables were then incorporated into multivariate Logistic regression analysis to determine the risk factors for intestinal failure occurring beyond 42 days postoperatively or death. A predictive nomogram was developed based on the results. Internal validation was performed using the bootstrap resampling method. For external validation, clinical data were collected from neonates with NEC treated during two separate periods: January 2016 to December 2017 and January 2024 to January 2025, which bracketed the primary training period. The performance of the nomogram was assessed using the receiver operating characteristic (ROC) curve, calibration curve, and decision curve analysis (DCA).

**Results:**

A total of 110 neonates with NEC were enrolled in this retrospective study. Among them, 21 developed the composite outcome of intestinal failure occurring beyond 42 days postoperatively or death. This group consisted of 5 cases of postoperative intestinal failure and 16 postoperative deaths. Among the deaths, 14 directly attributable to the progression of NEC and intestinal failure (such as enterogenic sepsis, refractory septic shock, progressive hepatic failure, etc.), one neonate died of severe respiratory infection, and one died due to the parental withdrawal of treatment. Multivariate logistic regression identified five independent risk factors significantly associated with the composite outcome: gestational age, history of asphyxia, multiple birth (twins), preoperative sepsis, and postoperative short bowel syndrome (*P* = 0.033, *P* = 0.016, *P* = 0.037, *P* = 0.015, *P* = 0.005). These variables were incorporated into a predictive nomogram, which demonstrated good discrimination with an area under the ROC curve (AUC) of 0.878 (95% CI: 0.804–0.952), a sensitivity of 90.5%, and a specificity of 80.9%. The external validation showed an AUC of 0.789 (95% CI: 0.632–0.947), with a sensitivity of 90% and a specificity of 66.7%, indicating good discrimination. Furthermore, both internal and external validation calibration curves showed moderate agreement between the predicted and actual outcomes, and DCA supported the model's clinical applicability.

**Conclusion:**

Gestational age, history of asphyxia, multiple birth, preoperative sepsis, and postoperative short bowel syndrome (SBS) were identified as key risk factors for intestinal failure occurring beyond 42 days postoperatively or death. The nomogram developed using these factors provided a quantitative, simple, and intuitive tool for clinical risk assessment of postoperative outcomes in patients with NEC.

## Introduction

Necrotizing enterocolitis (NEC) is a life-threatening gastrointestinal emergency in preterm infants, with postoperative mortality rates remaining as high as 20%–30%, while parenteral nutrition (PN)-dependent intestinal failure severely compromises long-term survival and quality of life (1, 2). Intestinal failure is defined as a condition where the intestinal function is reduced to the extent that it cannot meet the minimum requirements for the absorption of macronutrients, water and electrolytes, necessitating intravenous supplementation to maintain health and growth. In severe cases, it can lead to liver and kidney dysfunction, sepsis, and even death (3, 4). Although the American Society for Parenteral and Enteral Nutrition (ASPEN) guidelines define intestinal failure as PN dependence threshold beyond 60 days (5), The process of intestinal repair in neonates, especially preterm infants, exhibits distinct temporal dynamics: the intestinal epithelium completes its renewal cycle within 3–4 weeks (3). A postoperative interval of 42 days encompasses two full regenerative cycles, and persistent intestinal dysfunction beyond this period indicates exhaustion of regenerative potential. A postoperative interval of 42 days encompasses two full regenerative cycles. Therefore, persistent intestinal dysfunction beyond this period indicates an exhaustion of the gut's regenerative potential. Furthermore, when PN dependence exceeds 42 days, the risks of cholestasis and infection increase significantly (4). Given their limited metabolic reserves, preterm infants are highly susceptible to irreversible damage if intervention is delayed.

Building upon this mechanistic basis, this study innovatively proposed the concept of postoperative day 42 as a critical window period for intestinal functional recovery in neonates, particularly preterm infants. Using multivariate analysis, we identified independent risk factors associated with intestinal failure occurring beyond 42 days postoperatively or death, and developed a nomogram model to enable early risk stratification. This model not only supplemented the ASPEN criteria with neonatal-specific evidence but also aligned with the 42-day postoperative follow-up milestone in clinical practice. By facilitating earlier interventions, our findings established a methodologically robust and data-driven foundations for improving outcomes and guiding future research.

## Materials and methods

### Participants

Participants were selected from neonates diagnosed with NEC who underwent surgical treatment at the Surgery Department of Shanxi Children's Hospital between January 1, 2018 and December 31, 2023. The inclusion criteria were: (1) surgical confirmation of NEC and subsequent operative management; and (2) All surgeries were performed by the surgical team at the Children's Hospital of Shanxi Province. The exclusion criteria were: (1) Patients with incomplete clinical data; (2) Patients who did not undergo surgical treatment. This study was approved by the Ethics Committee of the Children's Hospital of Shanxi Province (IRB-WZ-2025-003), and informed consent was obtained from the families of all participating patients. Authors had no access to personally identifiable information during or after data collection; all data were anonymized prior to analysis.

### Data collection

The clinical data of participants were collected, including: (1) Birth conditions: gestational age, birth weight, gender, delivery mode, presence or absence of asphyxia history, whether twins, etc.; (2) Maternal pregnancy complications: whether the mother had gestational hypertension, gestational diabetes, premature rupture of membranes > 18 h, etc.; (3) Preoperative conditions and preoperative laboratory indexes: whether complicated by patent ductus arteriosus (PDA), presence or absence of brain injury (including intracerebral hemorrhage), preoperative white blood cell count, preoperative hemoglobin level, preoperative C-reactive protein (CRP) level; (4) Surgical methods; (5) Postoperative outcomes: Follow-up was conducted until 6 months after surgery to determine survival, whether the infant had intestinal failure, SBS, etc.

### Definition of outcome

The main outcome in this study was intestinal failure, which was defined as neonates with NEC who underwent surgical treatment and required continuous intravenous nutrition for more than 42 days after surgery (6).

### Rationale for selecting the composite endpoint of intestinal failure occurring beyond 42 days postoperatively or death

This study adopted the composite endpoint of intestinal failure occurring beyond 42 days postoperatively or death to provide a comprehensive assessment of the overall risk of severe adverse outcomes following NEC surgery. Prolonged PN dependence lasting more than 42 days is strongly correlated with death. The increased number of events enhances the statistical power of the analysis, thereby improving the model stability. Moreover, intestinal failure and death share common pathological pathways, such as dysregulated inflammatory responses and impaired mucosal repair. Death is the most severe and irreversible terminal outcome of partial intestinal failure. The early identification of intestinal failure provides a critical window for intervention, allowing clinicians to optimize infection control and nutritional strategies, thereby potentially reducing mortality risks. This dual-endpoint approach balances methodological rigor with clinical utility.

### Validation cohort

To externally validate the nomogram, a distinct temporal validation cohort was established to evaluate its generalizability across different time periods within our center. We enrolled neonates who underwent surgical intervention for NEC from two separate intervals: a historical cohort from January 2016 to December 2017 (prior to the training cohort) and a subsequent cohort from January 2024 to January 2025 (after the training cohort). All patients were selected by strictly adhering the same inclusion and exclusion criteria used for the primary training cohort, and all necessary clinical data were retrospectively and systematically extracted from the medical records.

### Statistical methods

Statistical analysis was conducted using R software (V.4.2.2) and SPSS software (V27.0), and statistical significance was set at *P* < 0.05. The continuous variables were presented as mean ± standard deviation (SD) or median and interquartile range (IQR, Q25−Q75). Continuous data between two groups were compared using the independent samples *t*-test for normally distributed data. In cases of skewed distribution, the Mann–Whitney *U*-test was employed. Furthermore, categorical data were compared using *χ*² or Fisher's exact test. Univariate analysis was conducted for all clinical variables, and potential predictors were incorporated into LASSO regression to identify the most relevant predictors. Variables with non-zero coefficients under lambda.1se were determined by 10-fold cross-validation method. Multivariate logistic regression was then used to identify independent clinical predictors of intestinal failure or mortality following NEC surgery. Based on the multivariate analysis results, a nomogram was developed. The model was internally validated by Bootstrap repeated sampling 1,000 times. The predictive value, consistency and clinical efficacy of the model were evaluated using the receiver operating characteristic (ROC) curve, calibration curve, and decision curve analysis (DCA).

## Results

### Comparison of general information

A total of 110 neonates with NEC were enrolled in this retrospective study. Among them, 21 neonates were classified into the composite outcome group of either intestinal failure occurring beyond 42 days postoperatively or death. This group included 5 cases of postoperative intestinal failure and 16 cases of postoperative death. Among the deaths, 14 were directly attributed to NEC progression and intestinal failure (such as enterogenic sepsis, refractory septic shock, progressive hepatic failure, etc.), one died of severe respiratory infection, and one followed parental decision to withdraw treatment. The remaining 89 neonates were classified into the non-intestinal failure group. No significant differences were observed between the two groups regarding gender, mode of delivery, birth weight, presence of PDA, preoperative brain injury, preoperative white blood cell count, hemoglobin level, surgical method, or whether the mothers had pregnancy complications (all *P* > 0.05). However, significant differences were found between the groups in twins, history of asphyxia, presence of preoperative sepsis, gestational age, postoperative SBS, and preoperative CRP level (*P* = 0.013, *P* = 0.002, *P* = 0.003, *P* = 0.005, *P* < 0.001, *P* = 0.042, as shown in Table 1).

**Table 1:** Comparison of clinical data between the group with intestinal failure or death after NEC surgery and the non-intestinal failure group.

Characteristics	Intestinal failure or death (*n* = 21)	Non-intestinal failure (*n* = 89)	*P* Value
Male [*N* (%)]	13 (61.9)	45 (50.6)	0.349
Spontaneous Delivery [*N* (%)]	7 (33.3%)	23 (25.8)	0.488
Twins [*N* (%)]	8 (38.1)	11 (12.4)	0.013
Maternal Complications during Pregnancy [*N* (%)]	11 (52.4)	58 (65.2)	0.276
Patent Ductus Arteriosus [*N* (%)]	9 (42.9)	41 (46.1)	0.790
Asphyxia History [*N* (%)]	11 (52.4)	17 (19.1)	0.002
Preoperative Brain Injury [*N* (%)]	7 (33.3)	41 (46.1)	0.290
Surgical Approaches [*N* (%)]			
Enterostomy	20 (95.2)	85 (95.5)	1.000
Anastomosis	1 (4.8)	4 (4.5)	
Postoperative Short Bowel Syndrome (SBS) [*N* (%)]	5 (23.8)	3 (3.4)	0.005
Low Birth Weight [N (%)]	18 (85.7)	57 (64.0)	0.055
Preoperative Sepsis [*n* (%)]	15 (71.4)	32 (36.0)	0.003
Gestational Age, Weeks (x ± sd)	32.02 ± 3.71	34.95 ± 3.16	<0.001
Preoperative White Blood Cell Count [×10^9^, Median, IQR]	5.01 (2.67,12.86)	7.07 (4.77,11.94)	0.156
Preoperative Hemoglobin, g/L (Median, IQR)	111 (98.50,137.50)	122 (107.50,148)	0.075
Preoperative CRP, mg/L (Median, IQR)	58.31 (28.06,87.13)	29.74 (8.40,67.40)	0.042

### LASSO regression for variable selection

**Figure 1:**
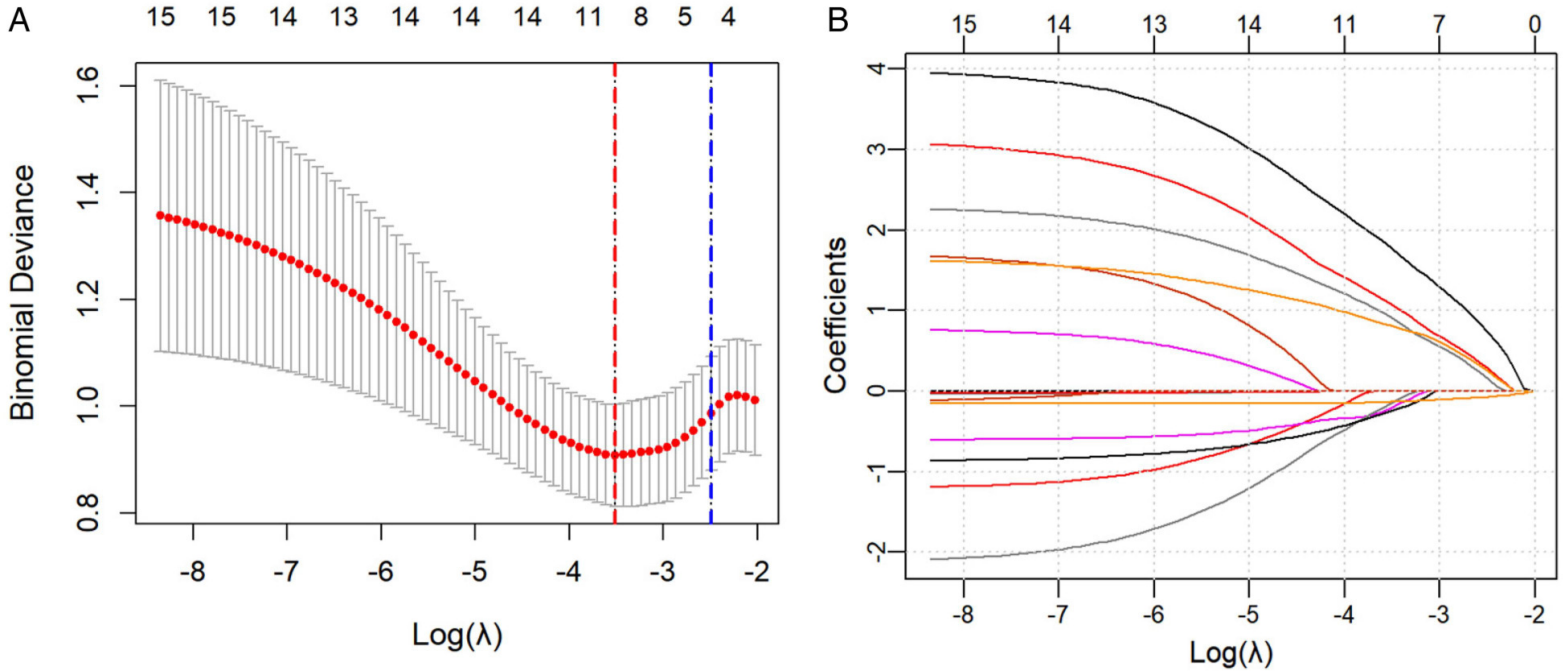
**(A)** A 10-fold cross-validation plot was drawn, with the red and blue dotted lines representing *λ*min [*λ* = 0.02972671, Log(*λ*) = −3.515709] and *λ*1se [*λ* = 0.08271633, Log(*λ*) = −2.492338], respectively. **(B)** LASSO Regression Coefficient Path Plot.

All potential predictive factors for severe adverse outcomes after NEC surgery were incorporated in the LASSO regression for variable selection. A 10-fold cross-validation plot was performed, with the red and blue dotted lines representing *λ*min [*λ* = 0.02972671, Log(*λ*) = −3.515709] and *λ*1se [*λ* = 0.08271633, Log(*λ*) = −2.492338]. The *λ*1se was selected as the optimal value (Figure 1). Ultimately, five variables with non-zero regression coefficients were identified:gestational age, history of asphyxia, twins, presence of preoperative sepsis, postoperative SBS (Figure 1).

### Multivariate logistic regression analysis of the occurrence of intestinal failure occurring beyond 42 days postoperatively or death

Multivariate Logistic analysis was conducted on the five predictive variables selected by LASSO regression. The results showed that gestational age, history of asphyxia, twins, preoperative sepsis, and postoperative SBS were independent risk factors for intestinal failure or death. (*P* = 0.033, *P* = 0.016, *P* = 0.037, *P* = 0.015, *P* = 0.005, as shown in Table 2).

**Table 2:** Multivariate logistic regression analysis of influencing factors for the occurrence of intestinal failure or death after NEC surgery.

Factor	B	SE	Wald	*P* Value	OR	95%CI
Twins	1.585	0.76	4.351	0.037	4.88	1.1–21.644
Asphyxia History	1.742	0.726	5.759	0.016	5.706	1.376–23.665
Postoperative SBS	3.015	1.077	7.839	0.005	20.387	2.47–168.253
Gestational Age	−0.225	0.105	4.546	0.033	0.799	0.65–0.982
Preoperativesepsis	1.67	0.685	5.95	0.015	5.31	1.388–20.313

### Nomogram development and scoring equation

**Figure 2:**
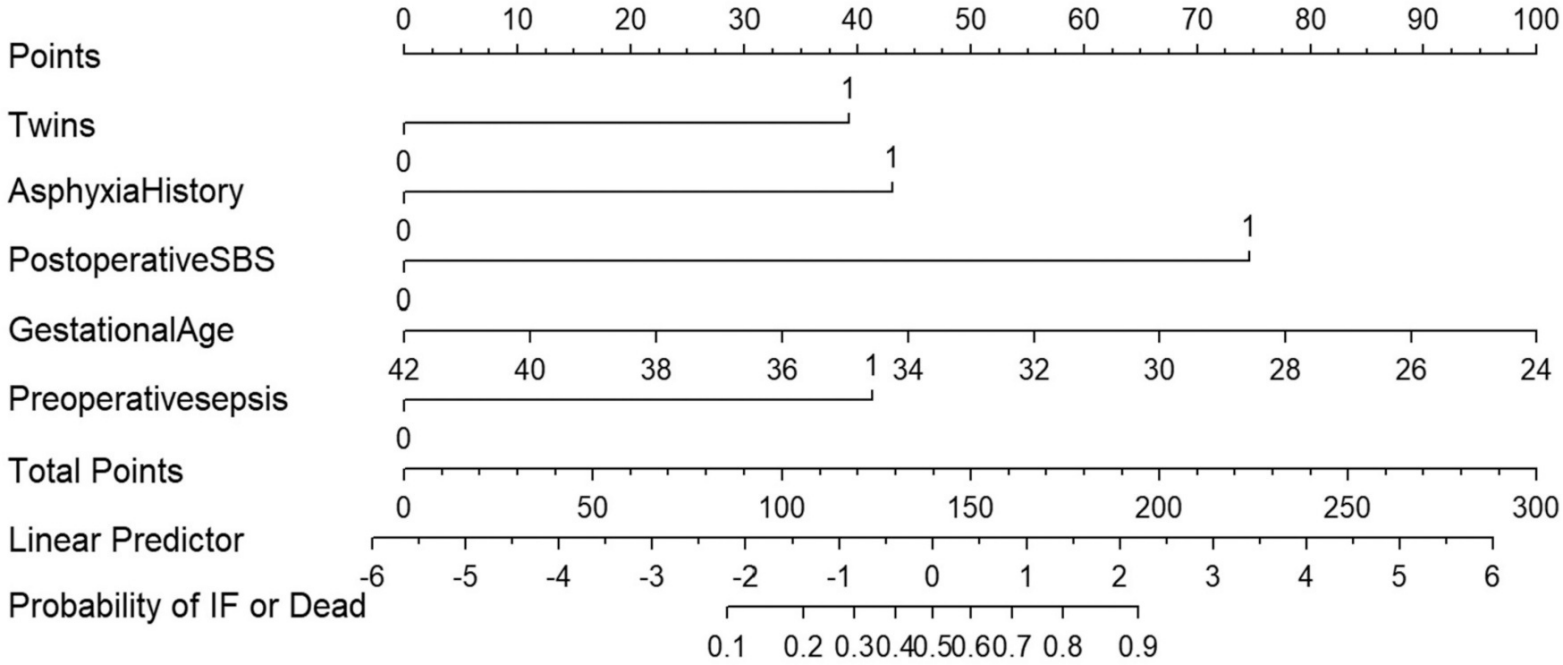
The nomogram model is used for predict the occurrence of intestinal failure or death after NEC surgery.

Based on the five variables identified by the multivariate Logistic regression analysis, a nomogram model was developed to predict the occurrence of intestinal failure or death after NEC surgery (Figure 2). The model demonstrated good discriminative ability, with a concordance index of 0.878 upon internal validation. To facilitate clinical application, we provide the scoring equation of the nomogram as follows:

logit(P)=3.775−0.225×GestationalAge+1.742×AsphyxiaHistory+1.585×Twins+1.67×Preoperativesepsis+3.015×PostoperativeSBS

*P* represents the predicted probability of the severe adverse outcome in neonates with NEC after surgery. *P* = e^logit(P)^/[1 + e^logit(P)^].

Each variable in the nomogram is assigned a score proportional to its predictive weight. The sum of these individual scores yields a total score, which directly corresponds to the probability of severe adverse outcomes following NEC surgery. For example, a singleton neonate born at 34 weeks without a history of asphyxia, yet presenting with preoperative severe sepsis and postoperative short-bowel syndrome, accumulated approximately 160 points, corresponding to a risk level of 68%. Surgeons should attach great importance to the neonate and take active intervention measures to improve the prognosis. a singleton neonate born at 34 weeks' gestation, with no history of asphyxia but presenting with preoperative severe sepsis and postoperative short-bowel syndrome, would accumulate approximately 160 points—corresponding to a 68% risk of severe adverse outcomes. Clinicians should prioritize such neonates and implement proactive interventions to optimize prognostic outcomes.

### Nomogram validation

**Figure 3:**
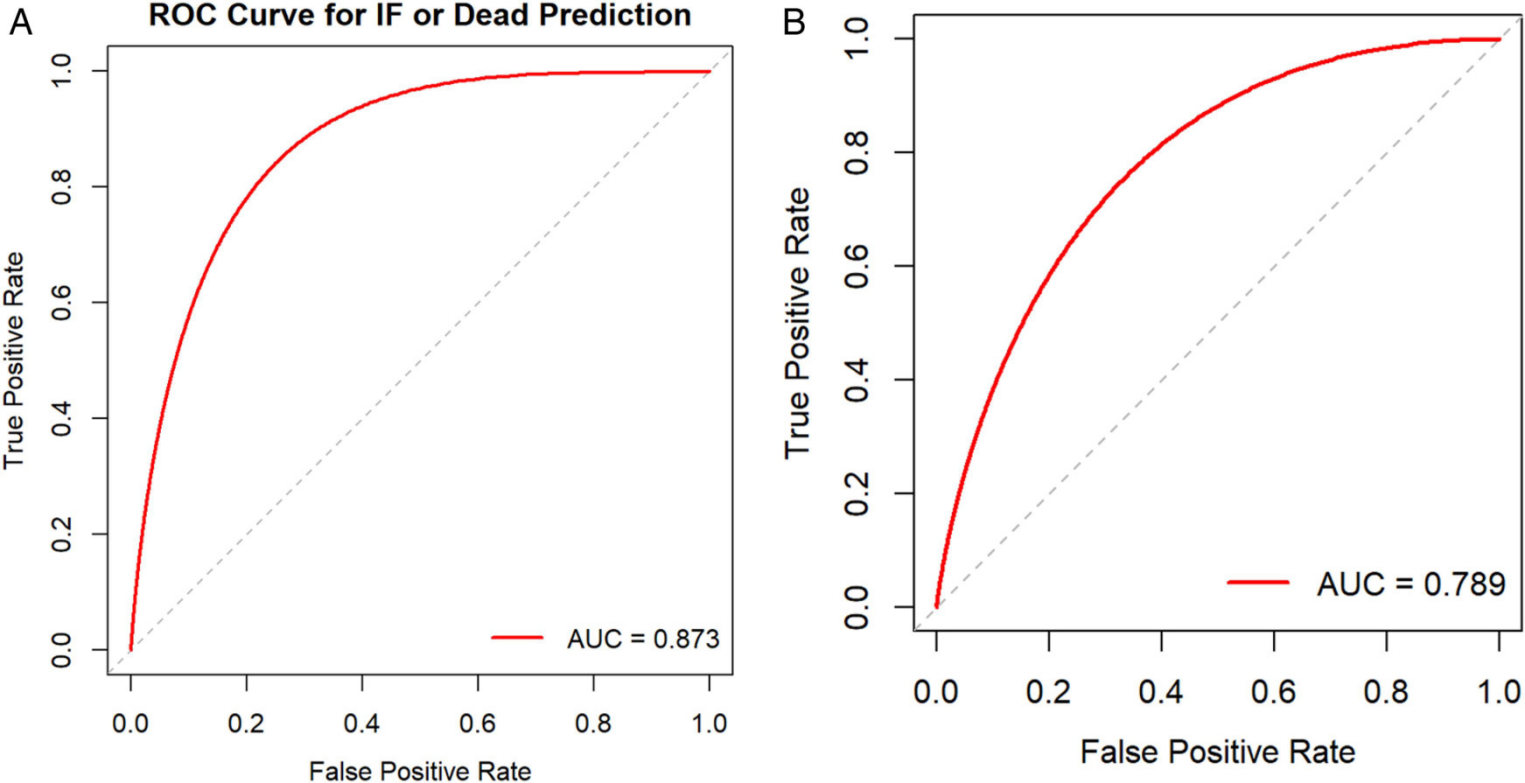
**(A)** Receiver operating characteristic curve (ROC curve) for the nomogram model. **(B)** External validation cohort ROC curve.

**Figure 4:**
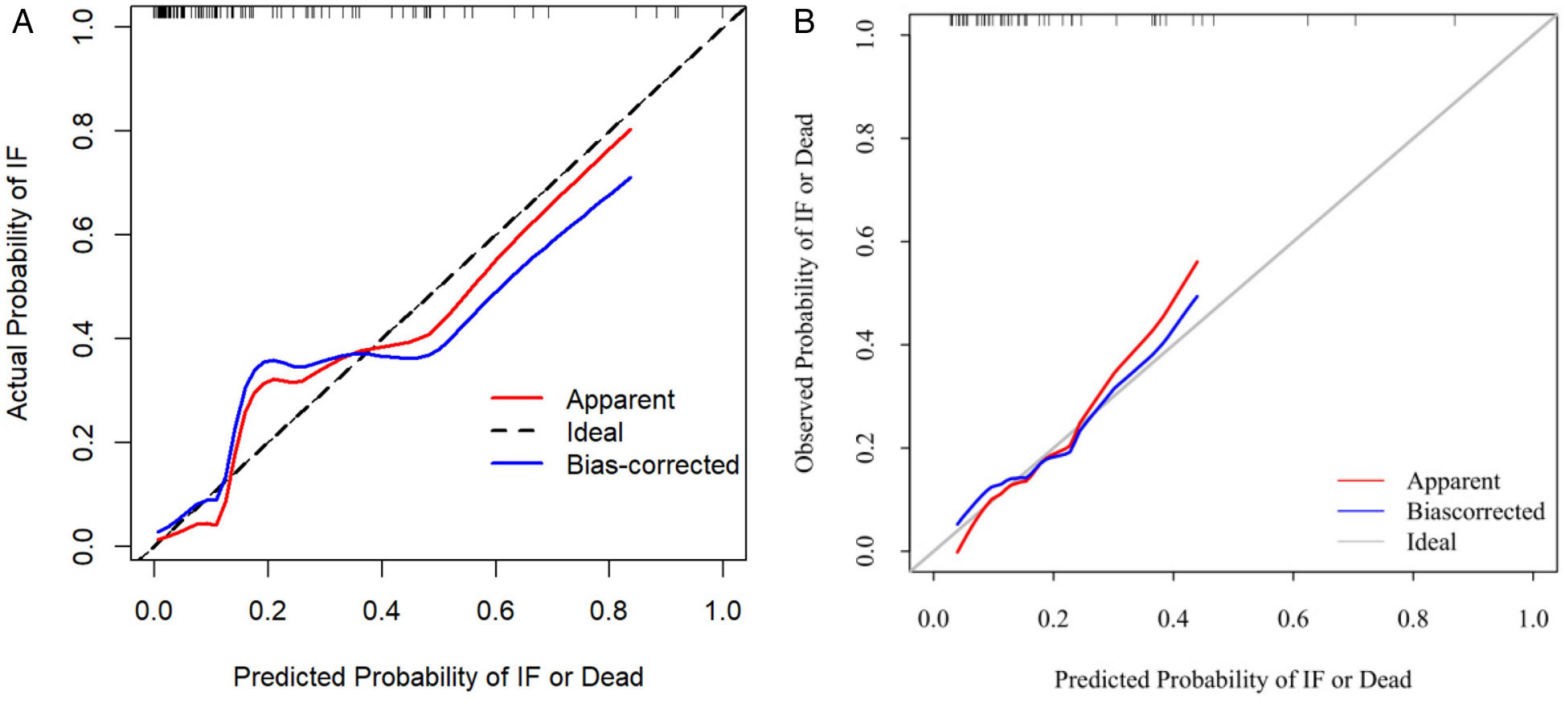
**(A)** Calibration curve of the model in the internal validation cohort. **(B)** External validation cohort Calibration curve.

**Figure 5:**
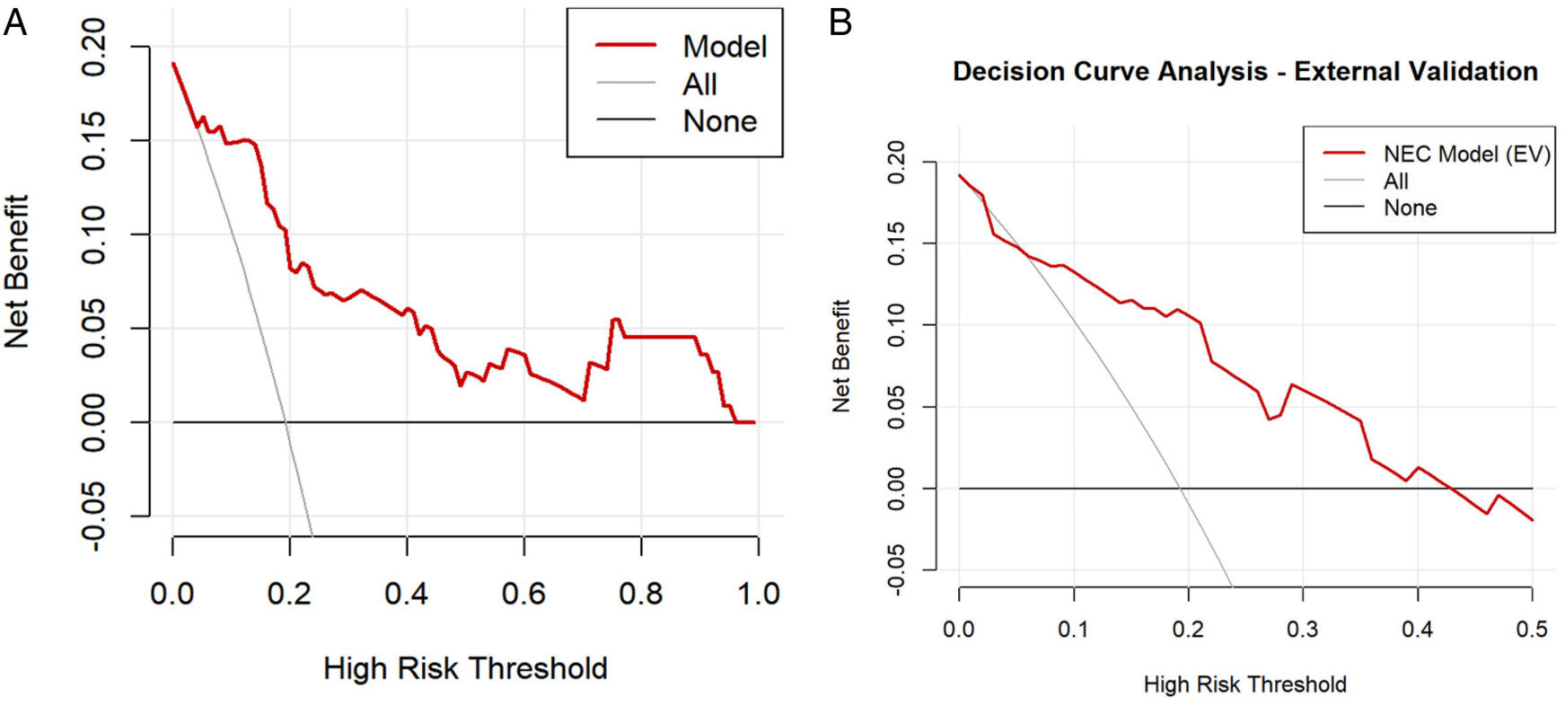
**(A)** Decision curve analysis (DCA) for the nomogram model. **(B)** Decision Curve Analysis (DCA) for the External validation.

The nomogram was internally validated using the bootstrap resampling method with 1,000 repetitions. The model demonstrated excellent discriminative ability, achieving an area under the curve (AUC) of 0.878 (95% CI: 0.804–0.952), with a sensitivity of 90.5% and a specificity of 80.9% (Figure 3A). The Hosmer-Lemeshow test yielded a *P*-value of 0.860, and the calibration curve exhibited high agreement between predictions and observations, indicating a moderatel agreement with the actual observed results (Figure 4A). Decision curve analysis (DCA) was performed to evaluate clinical utility, which showed a relatively high net benefit across a wide threshold probability range (0% to 95%), thereby supporting its potential for clinical application (Figure 5A).

Given the limited sample size of only 32 cases between January 2024 and January 2025, which was insufficient for robust model evaluation, we further expanded the cohort by including cases from an adjacent period (January 2016 to December 2017) to form a time-adjacent external validation set. This final external validation cohort enrolled 52 neonates with NEC who underwent surgical intervention. The model demonstrated good discriminative ability in this set, with an AUC of 0.789 (95% CI: 0.632–0.947), a sensitivity of 90%, and a specificity of 66.7% (Figure 3B). The Hosmer-Lemeshow test yielded a *P*-value of 0.845, indicating adequate model fit. Furthermore, the calibration curve showed good agreement between predicted probabilities and observed outcomes (Figure 4B). Decision curve analysis (DCA) further supported the model's clinical utility, demonstrating a favorable net benefit within a threshold probability range of 0% to 42% (Figure 5B).

## Discussion

Despite advancements in perinatal medicine that have reduced the overall mortality of NEC, a subgroup of critically ill neonates continues to experience persistently high rates of postoperative intestinal failure and mortality, presenting ongoing challenges in surgical management (7). While the 2021 ASPEN guidelines define intestinal failure as PN dependence exceeding 60 days (5), our study innovatively proposed postoperative day 42 as a disease-specific threshold for neonates, particularly preterm infants, based on their unique intestinal regenerative dynamics. First, the intestinal epithelial renewal cycle in neonates is approximately 3–4 weeks. A 42-day postoperative period thus encompasses two complete regenerative cycles, and persistent dysfunction beyond this point indicates a depletion of the intrinsic repair capacity. Furthermore, PN dependence beyond 42 days significantly increases the risks of cholestasis and sepsis. Given the limited metabolic reserves in preterm infants, any delay in intervention predisposes them to irreversible multiorgan injury. This refined threshold not only supplements the ASPEN criteria with neonatal-specific physiological evidence but also aligns with standard 42-day postoperative follow-up protocols, thereby facilitating earlier risk stratification and timely targeted interventions.

Gestational age, history of asphyxia, multiple birth (twins), preoperative sepsis, and postoperative SBS were identified as independent risk factors for intestinal failure occurring beyond 42 days postoperatively or death in neonates with NEC. Although these individual factors have been reported individually in previous studies, there remains a lack of a comprehensive, multivariate, and intuitive quantitative tool for risk assessment. To address this gap, this study further integrated and analyzed these five risk factors to develop a nomogram prediction model. In this model, each variable contributes a specific score, and the total score corresponds to a predicted probability of the composite outcome. As shown in the results, a 34-week gestational age singleton newborn without a history of asphyxia would have received a total score of approximately 160 points if diagnosed with preoperative sepsis and postoperative SBS, which indicates the probability of an adverse outcome was as high as 68%. This enabled rapid identification of high-risk patients for timely intervention.

Short bowel syndrome (SBS) is the most common cause of neonatal intestinal failure (8). After extensive intestinal resection, the remaining intestine in neonates with SBS will undergo a process of structural and functional adaptation. However, this adaptive response is often complicated by small intestinal bacterial overgrowth and malabsorption, which may lead to serious complications such as sepsis, malnutrition, and even death (9). Notably, NEC accounts for approximately 38.9% of SBS cases that require surgical resection of the intestine (10). For functional SBS caused by high-level enterostomy, effective reinfusion of intestinal fluids can promote mucosal growth and support intestinal adaptation, while also preventing atrophy of the distal bowel (11). In addition, an appropriate time for fistula closure based on the neonate's condition can also help prevent the occurrence of intestinal failure.

Prematurity and being a twin are significant factors contributing to poor prognosis in NEC. Adinda et al. reported that for NEC patients with a gestational age under 35 weeks, the postoperative mortality rate was 19%, with a complication rate of 57.1% (12). The intestinal mucosal barrier in these infants is often underdeveloped, heightening susceptibility to bacterial invasion and proliferation, which can exacerbate NEC lesions. Consequently, more extensive intestinal resections may be required, increasing postoperative mortality and prolonging intestinal recovery. Furthermore, premature infants inherently exhibit weaker intestinal motility and digestive capacity. Following NEC surgery, their intestinal function is further compromised and often make enteral nutrition insufficient to meet their growth requirements. As a result, most depend on parenteral nutrition support (13). However, prolonged parenteral nutrition can lead to intestinal mucosal atrophy, thereby increasing the risk of intestinal failure. Twin pregnancies are frequently associated with intrauterine growth restriction due to factors like elevated uterine pressure and insufficient placental function. This leads to higher rates of preterm birth and low birth weight compared to singleton pregnancies (14). Moreover, twins tend to have weaker immune function and a higher incidence of congenital malformations. These factors collectively contribute to a compromised tolerance for surgery, a greater need for postoperative intensive care, and a significantly increased mortality risk. Thus, both lower gestational age and twin gestation are important risk factors for an unfavorable surgical outcome in NEC.

Neonatal asphyxia triggers the diving reflex, leading to a redistribution of circulatory blood flow that prioritizes oxygen supply to vital organs. However, this process may result in intestinal ischemia and hypoxia, or even necrosis. Numerous studies have clearly reported that asphyxia is a key role in the development of NEC (15, 16), but its impact on NEC prognosis remains to be further investigated. Through multivariate Logistic regression analysis, this study identified that history of asphyxia as a risk factor for intestinal failure and death following NEC surgery (OR = 0.181, 95% CI: 0.043 to 0.765, *P* = 0.020), which may be associated with ischemia-reperfusion injury. While surgery can resect necrotic intestinal and halt the spread of infection, asphyxia impairs intestinal repair and disrupts normal functions, thereby inducing intestinal failure. Additionally, studies have suggested that neonatal asphyxia may disrupt the early colonization of intestinal microbiota, thereby exacerbating microbial imbalance and inflammatory responses while further increasing the risk of intestinal failure after NEC surgery (17). These infants are also more susceptible to postoperative complications such as anastomotic leakage, intestinal obstruction, and septic shock, all of which are critical contributors to postoperative mortality.

Infections and sepsis substantially impact the prognosis of NEC surgery. In this study, the incidence of preoperative sepsis in the intestinal failure or death group was 71.4%, significantly higher than the 36.0% incidence in the non-intestinal failure group. Similarly, the CRP level [58.31 mg/L (28.06, 87.13)] was also significantly elevated compared to that in the non-intestinal failure group [29.74 mg/L (8.40, 67.40)]. When sepsis and septic shock occur, the release of numerous inflammatory factors induces vascular endothelial damage, increased platelet consumption, and coagulation abnormalities, which can culminate in disseminated intravascular coagulation (DIC) and further exacerbating intestinal damage (18). Studies have demonstrated that platelet-activating factor is a key inflammatory mediator in NEC, which can cause intestinal epithelial cell damage and increased vascular permeability, thereby aggravating intestinal injury and increasing the risk of death (19, 20). Additionally, preoperative sepsis leaves infants with poor baseline health, characterized by low surgical tolerance, a high incidence of postoperative complications, and elevated mortality rates. Therefore, successful NEC surgery depends on the timely resection of necrotic bowel and thorough intraoperative irrigation and drainage. This approach not only helps control systemic inflammation and limit the extent of intestinal necrosis but also maximizes the preservation of viable bowel at the resection margin, effectively reducing the incidence of intestinal failure.

Furthermore, our etiological analysis of the fatal cases revealed that 5 neonates were diagnosed with intestinal failure, as they remained dependent on PN and other active treatments beyond 42 days postoperatively. Among the remaining 16 deaths, 14 were attributed to complications associated with NEC or intestinal failure, including enterogenic sepsis, refractory septic shock, and progressive hepatic failure. Another neonate's parents opted to withdraw treatment after 38 days of PN. Although this neonate did not strictly meet the diagnostic criteria for intestinal failure, he exhibited nearly all its objective features and would likely have fulfilled the definition had treatment continued. The final death resulted from severe respiratory infection. However, the underlying NEC that required 4 weeks of continuous PN postoperatively served as a major contributing factor. In summary, excluding 2 deaths indirectly related to intestinal failure, the remaining 19 cases (90.5%) clearly met the diagnostic criteria for either intestinal failure or death. This analysis demonstrates that intestinal failure and death are not independent competing outcomes, instead, death represents the most severe and irreversible consequence of intestinal failure in a subset of patients.

Regarding model validation, the nomogram demonstrated good discrimination in both internal (AUC = 0.878) and external validation (AUC = 0.789). Additionally, calibration curves indicated moderate agreement between predicted and observed outcomes. Decision curve analysis confirmed the model's significant clinical net benefit. Therefore, this visual scoring system allows clinicians to conduct rapid risk assessment and stratification in the early postoperative period, facilitating the identification of all high-risk infants prone to severe adverse outcomes. This approach can improve prognosis, substantially reduce mortality risk, and underscore the clinical utility of preemptive intervention, including personalized nutritional support, optimized intestinal management, and early initiation of intestinal rehabilitation therapy. Future work will focus on further refining and implementing this tool to enhance its clinical usability and accessibility (21).

## Limitations

This study has several limitations. First, it is a single-center, retrospective case-control study with a limited sample size. This may introduce bias and confounding factors, potentially restricting the model's predictive performance in larger populations. Second, while we used LASSO regression for variable selection, the low event-to-predictor ratio (21 events for 5 variables) was significantly below the recommended 10:1 guideline, which places the model at risk of overfitting and compromises the stability of the results. Furthermore, although internal bootstrap validation and a form of temporal validation were conducted, the model lacks true external validation from an independent, multi-center cohort. Consequently, the generalizability of our nomogram to other healthcare settings with different patient populations and clinical practices cannot be assured and requires future verification. Additionally, while our findings indicate that most fatalities were directly associated with NEC and intestinal failure, two deaths from other causes may have influenced the composite endpoint. Future large-scale, multi-center studies are needed to independently validate this model and analyze mortality specifically attributable to intestinal failure and NEC, thereby developing more accurate predictive models. We will also explore the integration of machine learning or deep learning techniques to enhance the model's predictive capabilities.

## Conclusion

This study identified gestational age, history of asphyxia, multiple birth (twins), preoperative sepsis, and postoperative SBS as significant risk factors for intestinal failure occurring beyond 42 days postoperatively or death in neonates with NEC. Based on the results of multivariate logistic regression, a simple and reproducible nomogram was developed to predict these outcomes. The model exhibits good discrimination ability and calibration, indicating considerable clinical potential. It can assist pediatric surgeons in early postoperative risk assessment, facilitating the implementation of individualized treatment strategies to prevent disease progression and improve prognosis for these patients.

## Data Availability

The original contributions presented in the study are included in the article/Supplementary Material, further inquiries can be directed to the corresponding author.
